# An enhanced light weight face liveness detection method using deep convolutional neural network

**DOI:** 10.1016/j.mex.2025.103229

**Published:** 2025-02-17

**Authors:** Swapnil R. Shinde, Anupkumar M. Bongale, Deepak Dharrao, Sudeep D. Thepade

**Affiliations:** a Department of Computer Science and Engineering, Symbiosis Institute of Technology, Pune Campus, Symbiosis International (Deemed University), Lavale, Pune, Maharashtra 412115, India; b Department of Artificial Intelligence and Machine Learning, Bharati Vidyapeeth Deemed to be University, Department of Engineering and Technology, Kharghar, Navi Mumbai, Maharashtra 410210, India; c Department of Artificial Intelligence and Machine Learning, Symbiosis Institute of Technology, Pune Campus, Symbiosis International (Deemed University), Lavale, Pune, Maharashtra 412115, India; d Department of Computer Engineering, Pimpri Chinchwad College of Engineering, Pune, India

**Keywords:** Biometrics authentication, Deep convolution neural network, Face spoofing detection, Light-weight architecture, LwFLNeT

## Abstract

Authentication plays a pivotal role in contemporary security frameworks, with various methods utilized including passwords, hardware tokens, and biometrics. Biometric authentication and face recognition hold significant application potential, albeit susceptible to forgery, termed as face spoofing attacks. These attacks, encompassing 2D and 3D modalities, pose challenges through fake photos, warped images, video displays, and 3D masks. The existing counter measures are attack specific and use complex architecture adding to the computational cost. The deep transfer learning models such as AlexNet, ResNet, VGG, and Inception V3 can be used, but they are computationally expensive. This article proposes LwFLNeT, a lightweight deep CNN method that leverages parallel dropout layers to prevent over fitting and achieves excellent performance on 2D and 3D face spoofing datasets. The proposed methods is validated through the Cross-dataset train test evaluation. The methodology proposed in the article has the following key contributions:

• Design of Light Weight Dual Stream CNN architecture with a parallel dropout layer to minimize over fitting issue.
• Design of Generalized and Robust deep CNN architecture that detects both 2D and 3D attacks with higher efficiency compared to existing methodology.
• Method validation done with State-of-the-Art methods using the standard performance metrics for face spoofing attack detection.

## Graphical abstract

**Figure:**
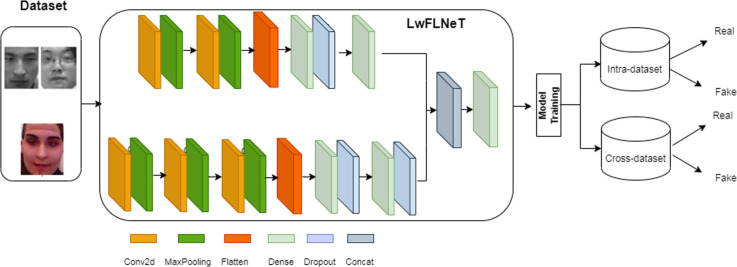

Specifications table

**Table:** 

Subject area:	Computer Science
More specific subject area:	*Biometrics Security, Deep learning*
Name of your method:	*LwFLNeT*
Name and reference of original method:	*NA*
Resource availability:	*3D MAD Dataset* https://www.idiap.ch/en/scientific-research/data/3dmad *Replay Attack Dataset* https://www.idiap.ch/en/scientific-research/data/replayattack *NUAA Imposter Dataset* https://parnec.nuaa.edu.cn/_upload/tpl/02/db/731/template731/pages/xtan/NUAAImposterDB_download.html *Code Repository* https://github.com/swapnilresearchHub/LwFLNet_Arch

## Background

Face spoofing detection is a critical component in securing face recognition systems against various attacks, including identity fraud and security breaches. As face recognition technology becomes increasingly widespread, the need for robust anti-spoofing measures has become more pressing [1]. While machine learning approaches have utilized texture-based and color space features for spoofing detection, they have failed to achieve satisfactory generalization performance. In contrast, deep learning-based approaches, particularly Convolutional Neural Networks (CNNs), have demonstrated excellent performance in detecting face spoofing attacks.

Recent studies have showcased the effectiveness of CNNs in learning discriminative features from face images to distinguish between genuine and spoofed faces. For instance, researchers have proposed CNN-based approaches that combine visual and depth features [2] and leverage multi-scale features [3,4] to improve detection accuracy. Other works have explored advanced CNN architectures, such as dense connection networks [5,6] and attention-based CNNs [7], to enhance detection performance. Additionally, a hybrid CNN-LSTM model has been introduced for face spoofing detection [8].

Despite these advancements, face spoofing detection remains a challenging task, particularly in scenarios involving high-quality display devices or print attacks. A recent study [9] proposed fine-tuned VGG-16 [10] and ResNet-50 [11] architectures that perform well for both 2D and 3D attack scenarios, outperforming existing state-of-the-art methods. However, this study only experimented on two datasets and did not address video replay attack scenarios. Most researchers have focused on evaluating their systems on either 2D or 3D datasets, with few designing robust systems that can detect both types of attacks. Those that have attempted to do so have only achieved satisfactory performance [12]. Therefore, further research is needed to develop more robust, reliable, and lightweight face spoofing detection systems that can generalize across various attack scenarios.

Pre-trained networks are often used as primary models with fine-tuning to address face spoofing attack detection. However, these models are typically heavy, with billions of parameters. There is a need for a deep CNN architecture that is lightweight [13], robust, and generalized for all forms of 2D attacks and 3D attack scenarios, including photo print, video replay, and mask attacks. This paper addresses these requirements and presents a robust and generalized model, comparing its performance on three publicly available spoof datasets and achieving higher results with respect to transfer learning architectures.

## Method details

The dataset used in the research article consists of 2D and 3D attack images obtained from different attack instruments. The description of each of the datasets is given below. After the datasets the article presents the explanation and working of proposed LwFLNeT architecture and other existing stat of the art methods.

3D MAD Dataset [14]: The 3D MAD dataset is the first publicly available 3D attack dataset with 76,500 images from 170 subjects, 1300 videos and 1300 attack images. Each individual 5 images for real and 5 images for attack are captured in different scenarios with light variations and expression variations. The images are captured using an Intel Real Sense F200 sensor, providing depth and RGB data. The dataset is mainly used to test the robustness of spoofing attack due to its variations. This dataset is made freely available on signing the EULA agreement with the authors/owners of the dataset. The Dataset is available as train, test and validation.

**Fig. 1:**
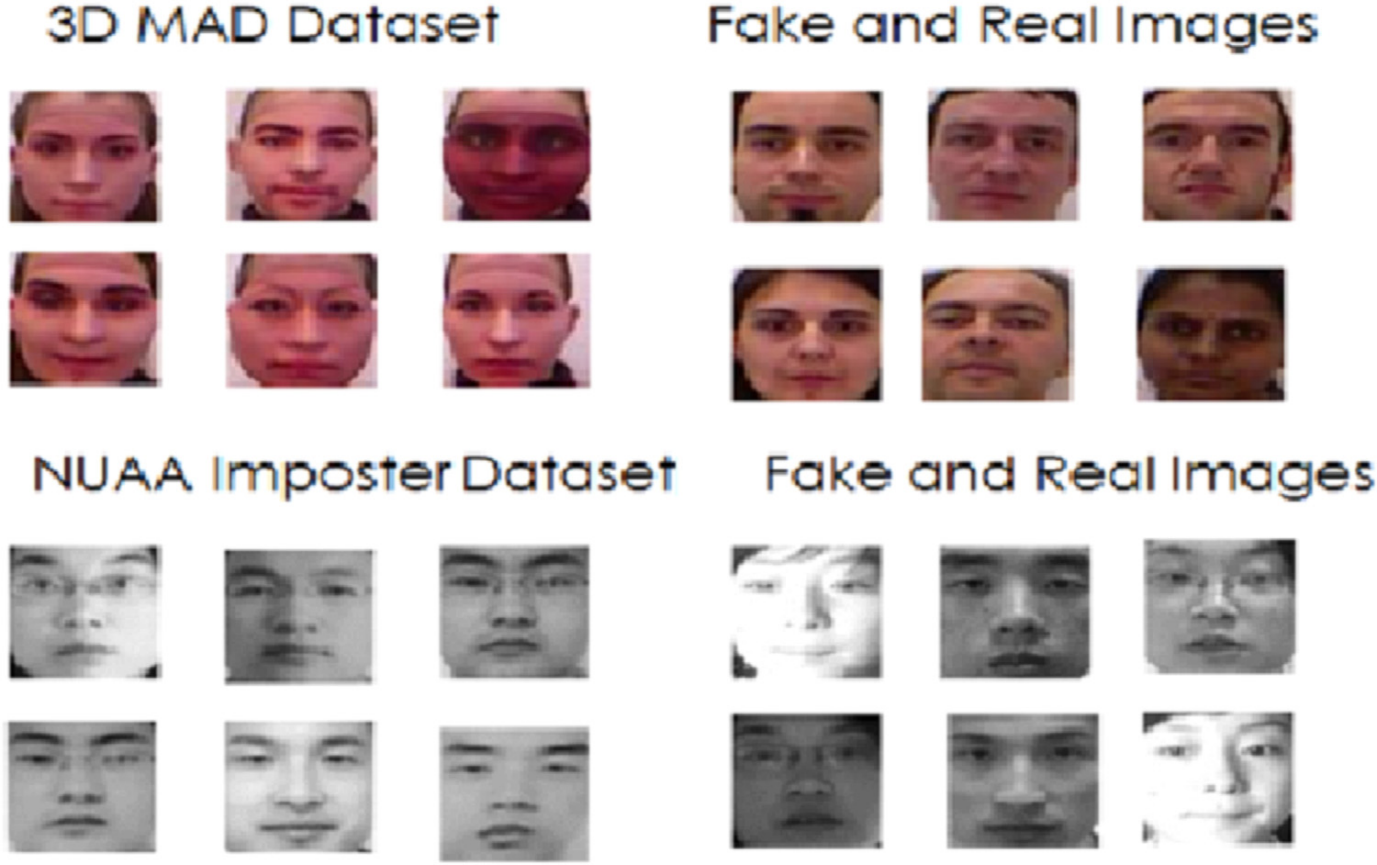
Sample images from 3D MAD and NUAA imposter dataset.

NUAA Imposter Dataset [15]: NUAA dataset consists of 5105 valid access images and 7509 presentation attack images. There are 10 real and 30 fake images for each subject in different light conditions and pose from different attack instruments such as printed photo, digital display and masks. The images are captured using high resolution camera with dimension of 1920x1080 pixels and then the face image is cropped and provided publicly for research. The sample dataset images for 3D MAD and NUAA are shown in Fig. 1 below.

**Fig. 2:**
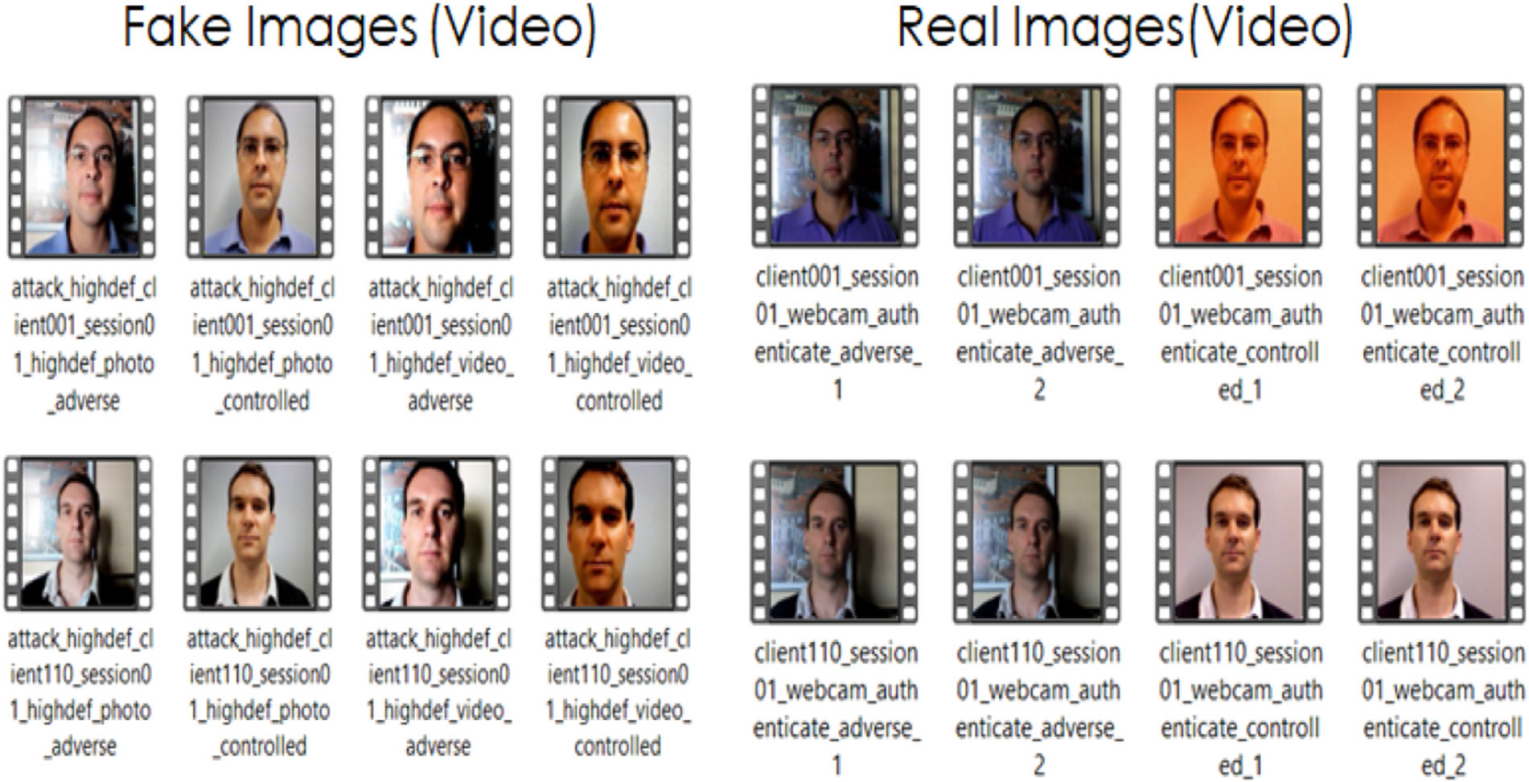
Sample images from video replay attack dataset.

Video Replay Attack Dataset [16]: It consists of 1300 video clips from 50 subjects of video and photo attack attempts. The dataset is captured under different lighting conditions using different attack instruments such as tablet or mobile phone or photo. The fake or real image is displayed for minimum 9 sec to the laptop to gain access to it. The sample dataset images for Video Replay attack dataset are shown in Fig. 2.

### Proposed light weight face liveness neural network (LwFLNeT)

**Fig. 3:**
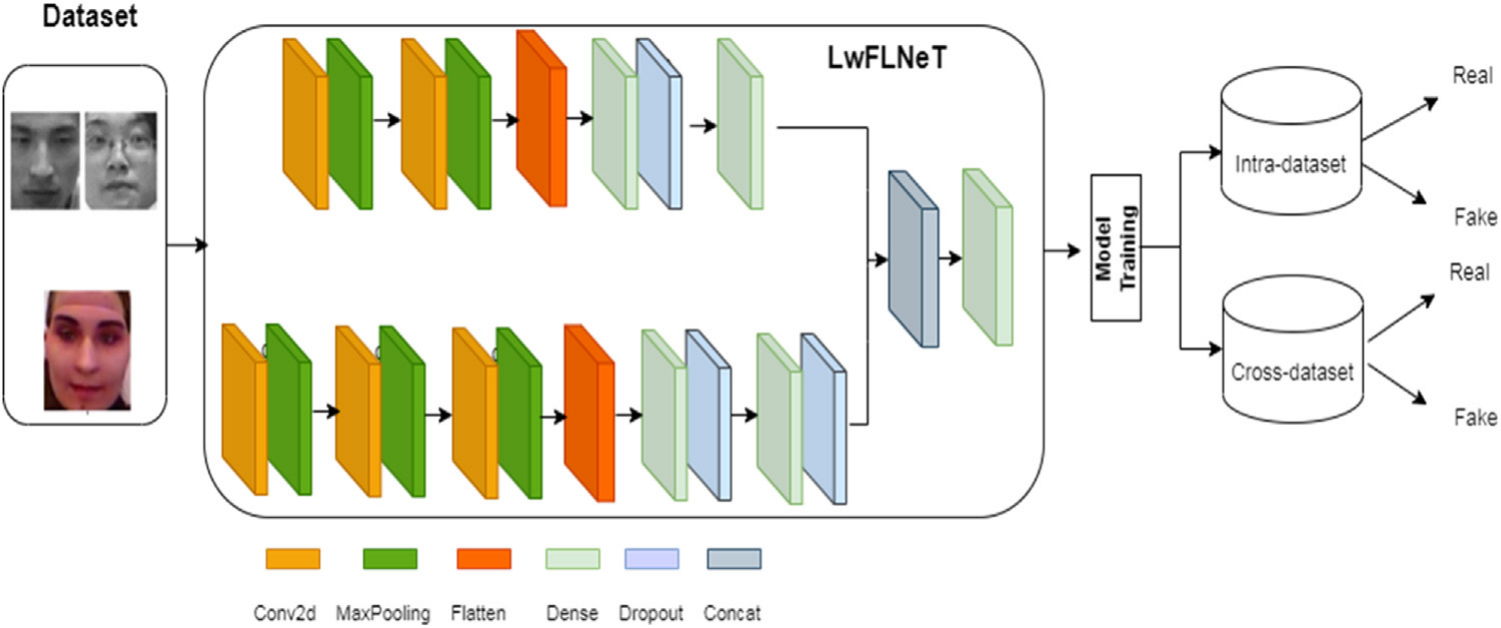
Generalized work flow for the proposed LwFLNeT architecture.

The proposed architecture is a light weight architecture compared to the state of the art methods. The general flow for the architecture is shown in the Fig. 3 below.

**Fig. 4:**
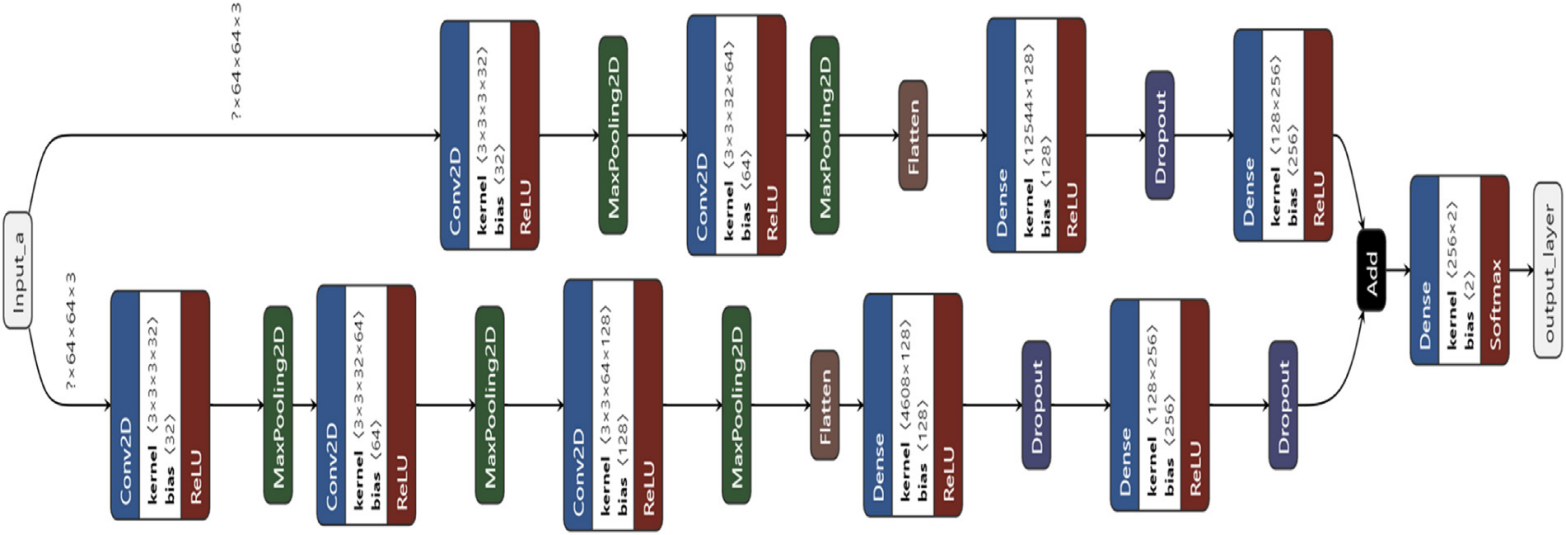
Proposed LwFLNeT architecture.

The proposed architecture layeerwise is shown in the below Fig. 4. The Architecture consists of a dual stream deep CNN architecture.

As can be seen in the above diagram the architecture is combination of Convolution [17] and Max pooling layers where there are three Convolution and Max pooling layer combinations on one stream of the architecture and two combinations on the other stream of the architecture. The convolution and max pooling sets are formed after multiple iterations, the kernel size and number of filters or each layer is the key contribution in the design of LwFLNeT architecture. There are multiple drop out layers [18] used in parallel in the dual stream architecture to reduce the effect of over fitting and achieve a higher performance in terms of the spoofing detection rate. The proposed architecture was tested on three datasets viz. 3D MAD [14], NUAA Imposter [15] and Video Replay [16] attack datasets, these three cover all forms of 2D and 3D face spoofing attack instruments. The Mathematical representation of the proposed model is given below in Table 1.

**Table 1:** Mathematical representation of the proposed LwFLNeT.

Step	Process
**Stream 1**	
Conv2D (32 filters, kernel size 3x3, ReLU activation):	$a_{1}=\varphi \left(Conv2D\left(x;W_{1}\right)+b_{1}\right)\;$
MaxPooling2D (2x2)	$a_{2}=Maxpool2D\left(a_{1}\;\right)$
Conv2D (64 filters, kernel size 3x3, ReLU activation):	$a_{3}=\varphi \left(Conv2D\left(a_{2};W_{2}\right)+b_{2}\right)\;$
MaxPooling2D (2x2):	$a_{4}=Maxpool2D\left(a_{3}\;\right)$
Conv2D (128 filters, kernel size 3x3, ReLU activation)	$a_{5}=\varphi \left(Conv2D\left(a_{4};W_{3}\right)+b_{3}\right)\;$
MaxPooling2D (2x2)	$a_{6}=Maxpool2D\left(a_{5}\;\right)$
Flatten	$a_{7}=Flatten\left(a_{6}\right)$
Dense (128 units, ReLU activation)	$a_{8}=\varphi \left(Dense\left(a_{7};W_{4}\right)+b_{4}\right)\;$
Dropout (0.4)	$a_{9}=Dropout\left(a_{8},0.4\right)$
Dense (256 units, ReLU activation)	$a_{10}=\varphi \left(Dense\left(a_{9};W_{5}\right)+b_{5}\right)\;$
Dropout (0.2)	$a_{11}=Dropout\left(a_{10},0.2\right)$
**Stream 2**	
Conv2D (32 filters, kernel size 3x3, ReLU activation)	$b_{1}=\varphi \left(Conv2D\left(x;W_{6}\right)+b_{6}\right)\;$
MaxPooling2D (2x2)	$b_{2}=Maxpool2D\left(b_{1}\;\right)$
Conv2D (64 filters, kernel size 3x3, ReLU activation)	$b_{3}=\varphi \left(Conv2D\left(b_{2};W_{7}\right)+b_{7}\right)\;$
MaxPool2D (2x2)	$b_{4}=Maxpool2D\left(b_{3}\;\right)$
Flatten	$b_{5}=Flatten\left(b_{4}\right)$
Dense (128 units, ReLU activation )	$b_{6}=\varphi \left(Dense\left(b_{5};W_{8}\right)+b_{8}\right)\;$
Dropout (0.4)	$b_{7}=Dropout\left(b_{6},0.4\right)$
Dense (256 units, ReLU activation)	$b_{8}=\varphi \left(Dense\left(b_{7};W_{9}\right)+b_{9}\right)\;$
Merge Stream 1 and 2	$a_{b}=\;a_{11}+\;b_{8}$
Final Layer	$Output=Softmax(Dense\left(a_{b\;};W_{10\;}\right)+\;b_{10}$

Let's denote the input tensor as x ∈ ℝ^(64x64x3).

The merged model takes the input x and passes it through both branches, then combines the outputs using element-wise addition. The final layer applies a softmax activation function to produce the output.

Note: φ represents the ReLU [19] activation function, and W_i_ and b_i_ represent the weights and biases for each layer, respectively.

The Stream 1 consists of multiple convolution layers that extracts complex features in terms of edges, textures, patterns, shapes, objects etc. In case of Face images the edges, shapes, textures and patterns are prominently displayed. On the other hand Stream 2 extracts high level features [20] with more dense layers. The combined output of both the models extract wide range of features that makes it generalized and robust to both 2D and 3D attacks.

The combination of the layers and inputs passed to each layer have been obtained after multiple iterations and then the drop out and classification layer have been added accordingly. The optimizer used is Adam optimizer with learning rate [21] of 0.0001 and batch size of 32. The Parameters used in the LwFLNeT architecture are shown in the Table 2 below. The Adam optimizer is most widely used in tasks related to image processing where complex high level features are extracted by the convolutional layers defined in the architecture.

**Table 2:** Parameters for LwFLNeT architecture.

Model Parameters	Value
Input shape	64x64x3
Filter size	3x3
Number of Blocks	17
Trainable Parameters	2374,914
Optimizer	Adam
Learning Rate	10–4

The Adam optimizer [22] performs a series of steps before reaching the convergence, it calculates the moving average gradient that represents the first moment (m1 at time w) . Then gets the second moment (m2 at time w) which is based on squared gradient at time w. These two undergo bias correction to obtain the new outputs m1_w_ and m2_w_. Finally, the convergence is adjusted based on the learning rate which updates the final output (n_t_) for learning rate (l). The respective equations for Adam are shown in (1), (2), (3), (4) and (5).

(1) $$m1=\beta 1*m1\left(w-1\right)+\left(1-\beta 1\right)*g_{w}$$

(2) $$m2=\beta 2*m2\left(w-1\right)+\left(1-\beta 2\right)*g_{w}^{2}$$

(3) $$m1_{w}=\;m1\;/\;\left(1-\beta 1^{w}\right)$$

(4) $$m2_{w}=\;m2\;/\;\left(1-\beta 2^{w}\right)$$

(5) $$n_{t}=m2\left(w-1\right)-l*m1_{w}/\sqrt{m2_{w}+\;\varepsilon }$$

where

m1 and m2 represent the first and second moment respectively.
$m1_{w}\;$represents first moment at time w, $m2_{w}$ is second moment at time w.
β1 and β2 represents first and second moment decay rate, g_w_ is the gradient at time w.
$n_{t}$ is the final output after convergence, ε – small value for numerical stability.

The above architecture yields 2M parameters which is quite low compared to the VGG-16 and RESNET-50 pre-trained and fine-tuned architecture.

### Existing state of the art methods

**Fig. 5:**
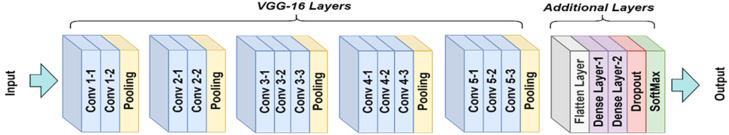
Fine-tuned VGG-16 architecture.

The Authors [9] have proposed fine-tuned architectures and presented results for the datasets viz. 3D MAD REPLAY and NUAA dataset. The most widely used VGG-16 and ResNet-50 architectures are fine-tuned and few layers have been added to enhance the performance of the existing architectures. The existing fine-tuned VGG-16 architecture is shown in below Fig. 5.

The output layer is replaced with a flatten layer and two dense layers followed by a drop out layer. The Dense layer 1 is defined with 4096 units and Dense layer 2 has 1072 units, The dropout applied to this architecture is 0.2. The Adam optimizer was used with a learning rate of 10–4. The loss function applied is sparse categorical cross entropy, optimizer used is Adam. All trainable layers are made false except the last three and trained on limited no of parameters. These were the modifications done to the existing VGG-16 architecture. The number of trainable parameters for the above fine-tuned architecture was 15 M which is very high and takes approx. more than 15 mins per epoch. The RGB colour space results were used for comparison with the proposed LwFLNeT architecture as this colour space extracts best results as per the author's analysis [23].

**Fig. 6:**
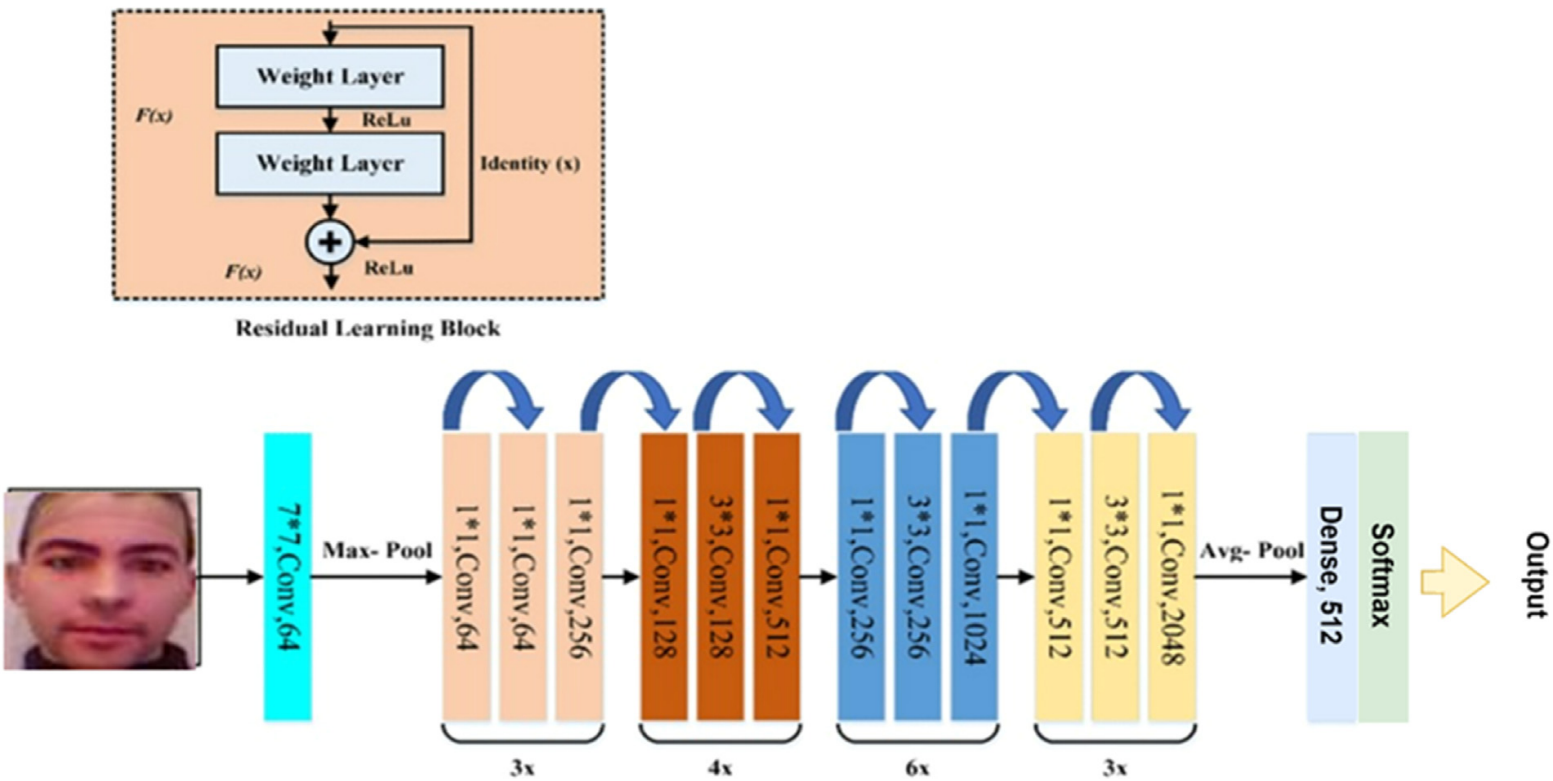
Fine-tuned RESNET-50 architecture.

The second existing fine-tuned architecture is the ResNet-50 architecture shown in below Fig. 6.

The fine-tuned RESNET-50 architecture output layer has a single dense layer with 512 units as compared to two dense layers in original architecture, followed by a classification layer with SoftMax classifier [24]. All trainable layers are made false except the last three and trained on limited no of parameters, yet the number of parameters is in millions. The above two existing architectures have been implemented based on the parameters mentioned by the authors [9] and are used for the comparison with the proposed LwFLNeT in terms of their standard error metrics used for spoofing detection. The comparison clearly indicates the generalization of the proposed architecture and its advantages over the existing complex and heavy architectures that have lower generalization capabilities for all the types of attack instruments.

## Method validation

The validation of the proposed LwFLNeT is done by comparing its performance with the existing state of the art methods mentioned in above section in terms of the standard metrics used for face spoofing detection. The standard metrics used for comparison are Accuracy, Bonafide Presentation Classification Error Rate (BPCER) [25,26], Attack presentation Classification Error Rate (APCER) and Half Total Error Rate (HTER) [27]. The formula for the metrics is given below. All these are based on the confusion matrix parameters.

Accuracy [28] – It's the ratio of correctly classified images in each class to the total number of images available in the dataset.

(6) $$\text{Accuracy}=\;\frac{\text{TP}+\text{TN}}{\text{TP}+\text{TN}+\text{FP}+\text{FN}}$$

Attack Presentation Classification Error Rate (APCER) – It's also termed as False Positive Rate (FPR), it denotes the number of fake images classified as real to the total number of images in the fake class.

(7) $$\text{APCER}=\;\frac{\text{FP}}{\text{TN}+\text{FP}}$$

Bonafide Presentation Classification Error Rate (APCER) – Its also termed as False Negative Rate (FPR), it denotes the number of real images classified as fake to the total number of images in the real class.

(8) $$\text{BPCER}=\;\frac{\text{FN}}{\text{TP}+\text{FN}}$$

Half Total Error Rate (HTER) – It's the mean of the FPR and FNR metrics. That indicates the overall performance of the proposed system in attack detection.

(9) $$\text{HTER}=\;\frac{\text{FPR}+\text{FNR}}{2}$$

The Result validation was performed on the three publicly available 2D and 3D attack datasets, the datasets were obtained through proper channel as per their procedure for usage. The results for the proposed method are shown in the below graphs, comparison of results for each proposed method in terms of dataset performance is shown for better understanding and analysis. 3D MAD, NUAA and REPLAY dataset results for VGG-16 and RESNET-50 performance parameters are obtained and compared with the proposed LwFLNeT architecture. The implementation was performed on a system with Core-i5 processor with 16GB of RAM and an SSD of 512MB and 4GB of GPU. The code was written on the Jupyter notebook where the LwFLNeT took less processing time compared to the fine-tuned models mentioned above.

**Fig. 7:**
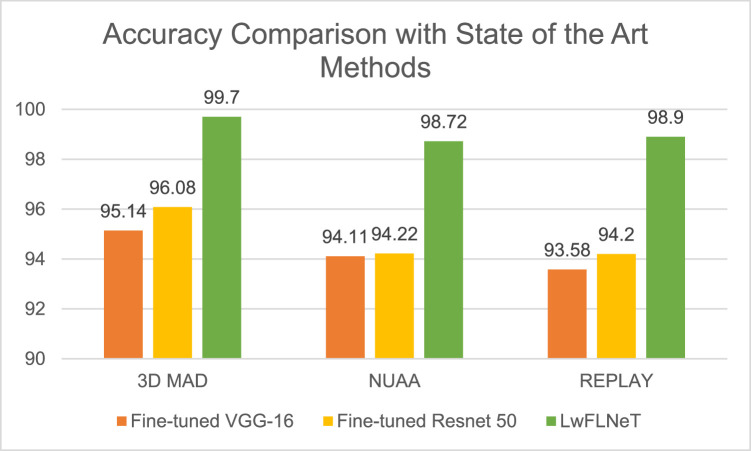
Accuracy comparison of proposed system with state-of-the-art methods for different datasets.

Results are shown in terms of different performance parameters with respect to the datasets used for validation. The below graph in Fig. 7 shows the comparison of the results in terms of accuracy for the 3D MAD dataset obtained by each of the methods/techniques.

The best accuracy on 3DMAD dataset is obtained by the LwFLNeT architecture as can be seen in above graph Fig. 7 which is 99.7 %, fine-tuned architecture obtained accuracy of 95.14 % for the VGG-16 at Epoch 30 for the RGB color space whereas 96.08 % for the RESNET-50 for the 3D MAD dataset. On NUAA dataset the best accuracy was obtained by the LwFLNeT architecture which is 98.72 % and other methods yielding an accuracy of 94 %. On the REPLAY dataset the LwFLNeT architecture achieved a promising accuracy of 98.9 % which was higher by 4–5 % compared to the existing methods.

**Fig. 8:**
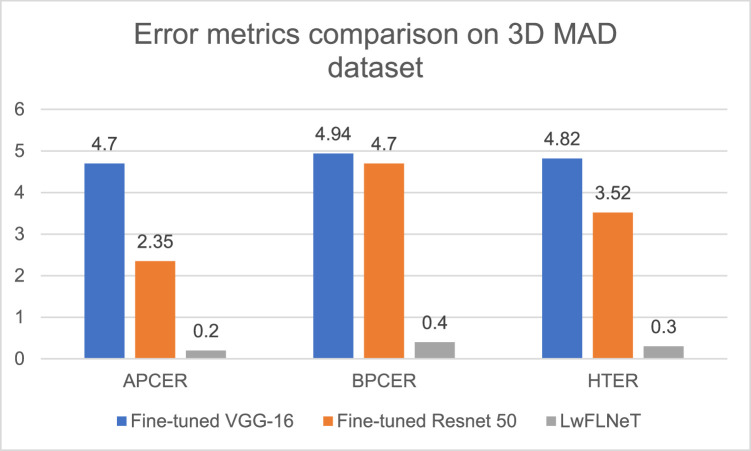
Error metrics comparison of proposed system with state-of-the-art method for 3D MAD dataset.

In terms of the error metrics the result comparison for 3D MAD dataset is shown in the Fig. 8.

The best APCER and BPCER obtained is 0.2 % and 0.4 % for the LwFLNeT. In terms of the fine-tuned networks the RESNET-50 achieves lowest value for APCER and BPCER compared to the VGG-16. LwFLNeT obtains the best HTER of 0.3 % which is best compared to the other state of the art methods.

**Fig. 9:**
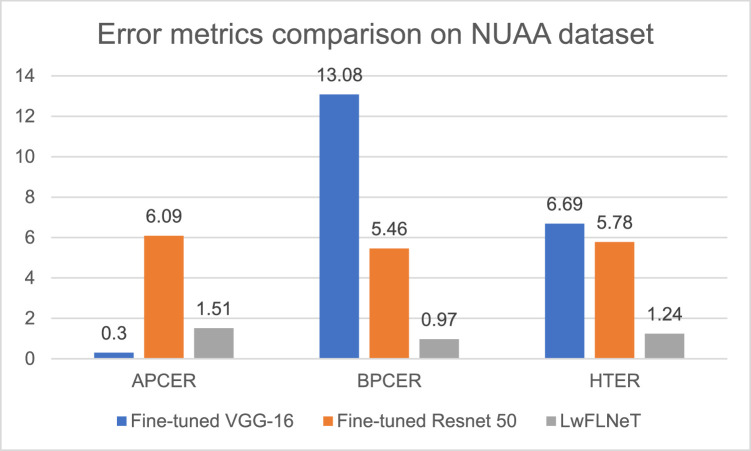
Error metrics comparison of proposed system with state-of-the-art methods for NUAA dataset.

The error metrics result comparison for NUAA dataset is shown in the Fig. 9 below.

The best APCER obtained is 0.3 % for the fine-tuned VGG-16 which is best among the three techniques and BPCER lowest value is for the LwFLNeT which is 0.97 %. LwFLNeT obtains the best HTER of 1.24 % which is best compared to the other state of the art methods.

**Fig. 10:**
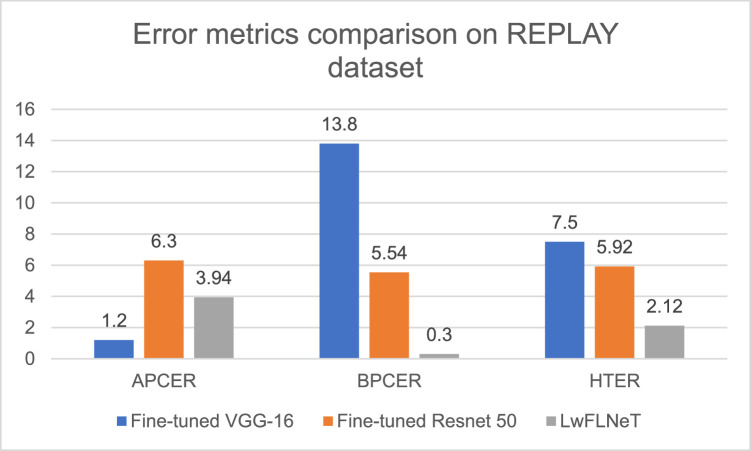
Error metrics comparison of proposed system with state-of-the-art methods for NUAA dataset.

The error metrics for the REPLAY dataset for all the three techniques is shown in the Fig. 10 below.

The best APCER obtained is 0.12 % for the LwFLNeT which is best among the three techniques and BPCER lowest value is for the LwFLNeT which is 3.12 %. LwFLNeT obtains the best HTER of 2.12 % which is best compared to the other state of the art methods.

The above accuracy results clearly indicate the generalization of the proposed LwFLNeT and its robustness to detect the 2D and 3D attack scenarios. Thus LwFLNeT outperforms the existing methods in the error metric analysis and has the best and lowest error rate. The objective of the proposed architecture was to design and validate a lightweight deep CNN architecture that's more robust and generalized for detection of 2D and 3D Face spoofing attacks. The above result comparison with the existing fine-tuned transfer learning models clearly indicate that the objective has been achieved and with a higher performance ratio.

The Inter dataset results comparison above shows that the proposed LwFLNeT architecture achieves the best results for both the 3D MAD, NUAA and REPLAY Dataset compared to the fine-tuned architectures.

Comparison Table for the same is shown below in Table 3.

**Table 3:** Comparison table for the architectures discussed in the article.

	Dataset	APCER (%)	BPCER (%)	HTER (%)	Accuracy (%)
Fine-tuned VGG-16	3D MAD	4.7	4.94	4.82	95.14
	NUAA	**0.3**	13.08	6.69	94.11
	REPLAY	1.2	13.8	7.50	93.58
Fine-tuned RESNET-50	3D MAD	2.35	4.7	3.52	95.14
	NUAA	6.09	5.46	5.78	94.22
	REPLAY	3.12	5.54	5.92	94.2
LwFLNet **(Proposed)**	3D MAD	**0.2**	**0.4**	**0.03**	**99.7**
	NUAA	1.51	**0.97**	**1.24**	**98.72**
	REPLAY	3.94	**0.3**	**2.12**	**98.9**

As can be seen in the above Table 3 the LwFLNeT architecture achieved the best accuracy of 99.7 % on the 3D MAD dataset, 98.72 % on NUAA dataset and 98.9 % on REPLAY dataset. Thus the proposed architecture surpasses the other models with a variation of 4, 5 % for 3D MAD and 4, 5 % for the NUAA Dataset and same can be observed for the REPLAY dataset. The above results show that the proposed architecture exhibits robust behavior in detection of video based attacks and image photo print attacks. Finally, we present the cross-dataset [29] validation for the proposed architecture which indicates the generalization of the proposed architecture in detection of any attack scenarios for 2D and 3D attacks. Table 4 below shows the cross dataset validation for two cases where in one case we trained using NUAA dataset and other case training was performed on the REPLAY dataset.

**Table 4:** Cross dataset results for proposed LwFLNeT architecture.

Parameters	TRAIN/TEST	TRAIN/TEST
Epoch 30	Case 1-NUAA/ 3D MAD	Case 2-REPLAY / 3D MAD
Accuracy	82.03	88.1
APCER	22.41	22.35
BPCER	14.28	6.67
HTER	18.34	14.5

The above Table 4 shows two scenarios where the training dataset was Replay Attack dataset and NUAA Imposter dataset, whereas the testing was done on the 3D MAD dataset. The proposed system achieves best cross dataset accuracy of 88 % which is quite excellent and satisfactory in terms of validation of the architecture. The Training done on the Replay Attack dataset yields very promising complex features combined with high level features that can generalize for any datasets showing an accuracy of 88 % and the error metrics obtained is also satisfactory. The NUAA trained set also showed satisfactory performance when tested on the 3D MAD dataset which achieved an accuracy of 82 %.

## Limitations

The proposed LwFLNeT has shown best results on the datasets used for evaluation, all the attack vectors have been covered in the three datasets. The 3D MAD dataset has few lightning variations applied in order to generate the dataset and no variations in terms of pose, background etc. are available. Research can be done on the newly proposed and publicly available datasets for 3D attacks in order to introduce more variations that can be leveraged by the system to train it on different 3D attack vectors for the real time use cases. The above system is a light-weight architecture mainly devised for the low-memory devices and tested on the publicly available standard datasets created in closed conditions, researchers can create custom real time datasets for face spoofing detection and test it with such light-weight architecture for their performance evaluation.

## Ethics statements

Ethical clearance is not applicable for the proposed work.

## CRediT author statement

**Swapnil R. Shinde:** Drafting the original article, methodology, implementation, and result analysis. **Anupkumar M. Bongale:** Drafting the original article, methodology, implementation, result analysis and supervision. **Deepak Sudhakar Dharrao:** Drafting the original article, methodology, implementation, result analysis and supervision. **Sudeep D. Thepade:** Drafting the original article, methodology, implementation, and result analysis.

## Funding

There is no funding for this research work.

## Declaration of competing interest

The authors declare that they have no known competing financial interests or personal relationships that could have appeared to influence the work reported in this paper.

## Data Availability

Data is available upon a reasonable request.
